# Unmet health care needs for patients with thyroid disease including thyroid cancer among Bangladeshi population

**DOI:** 10.7189/jogh.15.03042

**Published:** 2025-10-24

**Authors:** Abidur Rahman, Syed Emdadul Haque, Yurie Kobashi, Masashi Tomioka

**Affiliations:** 1 Institute for Professional Excellence & Research, Micare Health, Dhaka, Bangladesh; 2 UChicago Research Bangladesh, Dhaka, Bangladesh; 3 Department of Laboratory Medicine, Fukushima Medical University, Fukushima, Japan; 4 Global Exchange Center, Fukushima Medical University, Fukushima, Japan; 5 Baptist Mid-Mission Hospital, Natore, Bangladesh

## Abstract

We aimed to identify the unmet health care needs of and proposed potential solutions for patients with thyroid diseases, including those with thyroid cancer, among the Bangladeshi population. We performed a qualitative study using structured interviews with healthcare professionals involved in care management and surgery for thyroid diseases. The interviews were conducted in four hospitals across three large cities in Bangladesh, with participants gathered through convenience sampling. Key findings were categorised and summarised into themes by topic, such as challenges, barriers, unmet needs, and solutions. The most common triggers for visiting the hospital were thyroid (neck) swelling and palpitations. All facilities mentioned thyroid patients’ delayed arrival at the hospital and delayed diagnosis as problems, while incomplete treatment, incomplete follow-up, and inadequate specialised medical care emerged as challenges among thyroid cancer patients, specifically. Solutions to each issue and unmet need among thyroid patients focussed on improving awareness of thyroid disease, with raising awareness being the most important solution.

Optimal healthcare delivery, encompassing attitudes toward health, healthcare costs, health disparities, needs assessments, professional practice gaps, and overdiagnosis, is a crucial global health issue. An estimated 586 202 cases of thyroid cancer were reported in 2020, making it the 10th most common cancer worldwide [1]. Overdiagnosis of thyroid cancer is a particularly major problem in high-income countries; for instance, some countries reported no overdiagnosis, while others reported it to occur in 85% of cases [2]. Under these circumstances, careful assessment of needs is a vital for patients with thyroid cancer. Yet this is extremely challenging to do, especially in low- and middle-income countries (LMIC) which lack adequate data and have weak healthcare systems. According to Tieulent and colleagues, overdiagnosis is already a major problem in some LMICs, while in others such has India and Uganda, the incidence rate of thyroid cancer was reported to be low, while the exact mortality rate remains unknown [3]. There also remain crucial issues regarding the disparities between urban and rural areas [3]. In particular, cancer incidence has increased in Asia in recent years, owing to population growth, improved life expectancy, and a transition to an urban lifestyle [4].

Despite this, there is no information on the unmet needs in the community of patients with thyroid cancer in Asian LMICs. Here, we understand unmet needs as any barriers to receiving healthcare during the course of disease progression, such as lack of public awareness, high costs, unavailability of resources, limited access to specialised centers, and inadequate follow-up procedures.

Bangladesh, a South Asian country with an estimated population of approximately 175.7 million, struggles with socioeconomic challenges and a resource-constrained health system [5]. While the precise burden of thyroid disorders in Bangladesh is not known, with a community-based survey conducted at the district level suggesting that approximately 20% of the general population is affected [6], it still presents a major public health challenge. Over recent years, the incidence of thyroid cancer, especially papillary thyroid carcinoma, has increased gradually, although the mortality rate remains low [7]. The malignancy incidence of solitary thyroid nodules was observed to be significantly higher in Bangladesh (18.65%) than in other countries [7], with patients commonly presenting to the surgical outpatient department with this disorder. Approximately 8% of the adult population has clinically palpable nodules of the thyroid gland [8]. With advances in imaging techniques, especially high-resolution ultrasonography, the rates of detection of clinically impalpable thyroid nodules have increased manifold [9,10].

Considering these issues and research findings, we aimed to identify the unmet healthcare needs of and proposed potential solutions for patients with thyroid diseases, including thyroid cancer, among the Bangladeshi population.

## INTERVIEWS WITH HEALTHCARE PROFESSIONALS MANAGING PATIENTS WITH THYROID DISEASE

**Figure 1:**
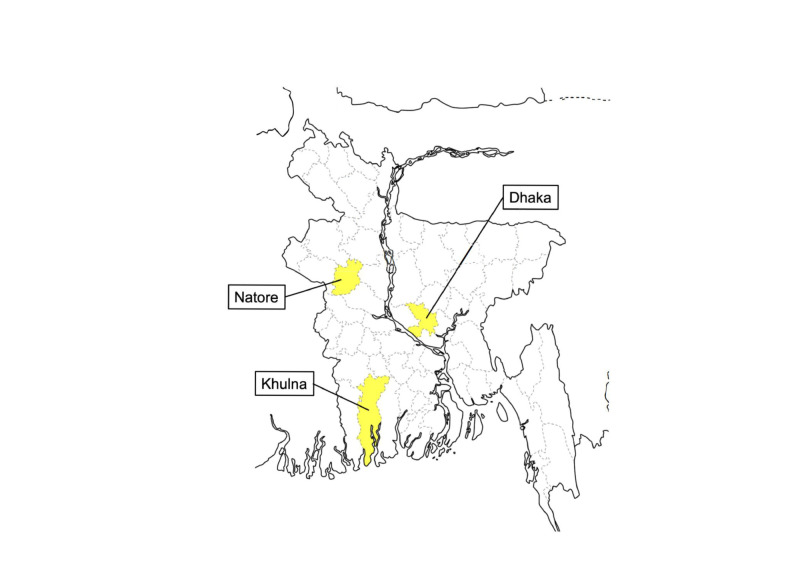
Map of study site. Interview survey was performed in hospitals in yellow highlighted prefectures

We performed a qualitative study based on structured interviews with healthcare professionals involved in care management and surgery for thyroid diseases. The interviews were conducted in four hospitals across three large cities in Bangladesh: Dhaka, Khulna, and Natore (**Figure 1**), with participants gathered through convenience sampling.

### Participants

We enrolled endocrinologists, thyroid specialists, surgeons performing thyroid surgeries, oncologists managing patients with thyroid cancer, and hospital administrators overseeing patient care and facilities from four hospitals in Bangladesh. These individuals were selected based on their capacity to perform thyroid surgeries and their role in regional healthcare.

### Questionnaire

Local researchers conducted all interviews through paper-based questionnaire with open-ended questions or through face-to-face interviews. To avoid an interview bias, the researcher who prepared the interview guide did not conduct the interviews. The aforementioned interview guides covered the following key areas:

– common symptoms prompting patients to seek hospital care;

– major causes leading to thyroid surgeries;

– barriers and delays in diagnosis and treatment;

– availability and accessibility of diagnostic facilities;

– challenges in patient follow-up and continuity of care;

– existing gaps in specialised medical care for thyroid patients;

– proposed solutions and recommendations to improve patient care and outcomes.

### Data collection and analysis

Our qualitative analysis, performed by one author (YK), followed Braun and Clarke’s thematic analysis approach [11], where we categorised and summarised key findings into themes by topic, such as challenges, barriers, unmet needs, and solutions. The interviews lasted between 30 to 60 minutes.

### Ethical considerations

The Fukushima Medical University waived the ethics review process, as the study was considered exempt from Japan’s Ethical Guidelines for Medical and Biological Research Involving Human Subjects (reference number: REC2024-232).

## FINDINGS

An average of 28 (median of 30) thyroid surgeries were performed at each hospital per month.

### Triggers for hospital visits

The most common triggers for visiting the hospital were thyroid (neck) swelling and palpitations. All facilities mentioned encountering the symptoms of thyroid (neck) swelling among patients. Other symptoms included weight loss, weight gain, excessive sweating, changes in voice, difficulty swallowing, respiratory distress, intolerance to cold or heat, and tiredness.

### Surgery causes

The most common reasons for thyroid surgery were nodular goiter (approximately 77%), papillary carcinoma (approximately 10%), Hashimoto’s thyroiditis, and follicular adenoma. Three of the four interviewed hospitals reported the proportion of diagnostic diseases associated with surgery.

### Prioritised issues

All facilities mentioned that delayed diagnosis, defined as the time between the onset of symptoms and clinical diagnosis, was a common problem among thyroid patients in general. Among patients with thyroid cancer specifically, incomplete treatment, incomplete follow-up, and inadequate specialised medical care emerged as important issues. Notably, the staff reported that autoimmune antibody assays (TRAb, TPO-Ab, Tg-Ab, and Tg) were not conducted in thyroid patients regularly. The schedule of follow-up duration for thyroid cancer patients differed among facilities, and examinations of metastasis were not conducted by some of the surgeons in district hospitals.

### Solutions

Solutions to each issue and unmet need among thyroid patients focussed on improving awareness of thyroid disease, describing proper steps of thyroid treatment, counselling the patients about the follow-up procedure, and increasing the number of radio-imaging facilities at the district level. Raising awareness emerged as the most important solution for both thyroid diseases overall and thyroid cancer specifically. Other examples included increasing the number of operations, financial support, and specialised thyroid centers at the district level.

## IMPLICATIONS

We interviewed four hospitals that performed thyroid surgery in four districts in Bangladesh regarding unmet needs, issues, and solutions for patients with thyroid diseases, including thyroid cancer.

The most common trigger for visiting the hospital in patients with thyroid cancer was thyroid swelling, as mentioned by all facilities. Previously, individuals from iodine-deficient regions commonly presented with goitres; however, goitre prevalence is not only caused by iodine sufficiency [7]. Self-examination of thyroid swelling should be conducted properly for the early detection of thyroid disorders.

Benign nodules may be a major indication for thyroid surgery. The most common reason for thyroid surgery was nodular goitre, while papillary carcinoma was observed in approximately 10% of cases in our study. The incidence of papillary thyroid carcinoma has been steadily increasing over the decades in Bangladesh [5]. Standard and quantified diagnostic centres with histopathological facilities are crucial for the adequate diagnosis of thyroid cancer.

The issues among patients with thyroid disease could not be solved only within the hospitals, as they all mentioned that patients delayed their arrival at the facility and their diagnosis. While patients are increasingly being diagnosed during early phases of thyroid disorders, a significant portion of the population remains underdiagnosed. Community-level programmes should therefore be implemented by both governments and private organisations. The facilities in our study mentioned that raising awareness in the community was the most important solution for both thyroid disease and cancer. This would mean that focussing on health education among community members, as well as strengthening general access to healthcare services, is key for earlier diagnosis and treatment of thyroid disorders.

We note some limitations of this study. First, the interviews were conducted primarily among urban populations; future research should extend to remote and rural areas to ensure better representation across regions. Second, the healthcare professionals reported on their own experiences with treating thyroid cancer patients, which may not fully reflect objective clinical data. Third, our findings are specific to a particular context and individuals with specific demographic backgrounds, meaning they may not generalise to other settings with differing infrastructures, economic constraints, or patterns of healthcare access. Fourth, we selected the study hospital and interview participants with convenience sampling, which might have caused selection bias. Fifth, the proposed solutions were based on the participants’ perceptions of patient needs, not on evidence. Despite these limitations, we note that this is the first study to survey the unmet needs of Bangladeshi patients with thyroid diseases.

In conclusion, the most common trigger for visiting the hospital in patients with thyroid cancer was thyroid swelling, while interviewees from all facilities mentioned that thyroid patients delayed their visit to the hospital and diagnosis. A well-designed community-level programme should be implemented to provide healthcare for patients with thyroid cancer.
